# The spatial distribution of tree–tree interaction effects on soil microbial biomass and respiration

**DOI:** 10.1002/ece3.11530

**Published:** 2024-06-18

**Authors:** Henriette Christel, Helge Bruelheide, Simone Cesarz, Nico Eisenhauer, Georg J. A. Hähn, Rémy Beugnon

**Affiliations:** ^1^ German Center for Integrative Biodiversity Research (iDiv) Halle‐Jena‐Leipzig Leipzig Germany; ^2^ Institute of Biology, Leipzig University Leipzig Germany; ^3^ Institute of Biology/Geobotany and Botanical Garden, Martin Luther University Halle‐Wittenberg Halle Germany; ^4^ BIOME Lab, Department of Biological, Geological and Environmental Sciences (BiGeA) Alma Mater Studiorum University of Bologna Bologna Italy; ^5^ Institute for Meteorology, Leipzig University Leipzig Germany; ^6^ CEFE, Univ Montpellier, CNRS, EPHE, IRD Montpellier Cedex 5 France

**Keywords:** BEF‐China, belowground overyielding, biodiversity–ecosystem functioning, soil microbial biomass, subtropical forest, tree–tree interactions

## Abstract

The capacity of forests to sequester carbon in both above‐ and belowground compartments is a crucial tool to mitigate rising atmospheric carbon concentrations. Belowground carbon storage in forests is strongly linked to soil microbial communities that are the key drivers of soil heterotrophic respiration, organic matter decomposition and thus nutrient cycling. However, the relationships between tree diversity and soil microbial properties such as biomass and respiration remain unclear with inconsistent findings among studies. It is unknown so far how the spatial configuration and soil depth affect the relationship between tree richness and microbial properties. Here, we studied the spatial distribution of soil microbial properties in the context of a tree diversity experiment by measuring soil microbial biomass and respiration in subtropical forests (BEF‐China experiment). We sampled soil cores at two depths at five locations along a spatial transect between the trees in mono‐ and hetero‐specific tree pairs of the native deciduous species *Liquidambar formosana* and *Sapindus saponaria*. Our analyses showed decreasing soil microbial biomass and respiration with increasing soil depth and distance from the tree in mono‐specific tree pairs. We calculated belowground overyielding of soil microbial biomass and respiration – which is higher microbial biomass or respiration than expected from the monocultures – and analysed the distribution patterns along the transect. We found no general overyielding across all sampling positions and depths. Yet, we encountered a spatial pattern of microbial overyielding with a significant microbial overyielding close to *L. formosana* trees and microbial underyielding close to *S. saponaria* trees. We found similar spatial patterns across microbial properties and depths that only differed in the strength of their effects. Our results highlight the importance of small‐scale variations of tree–tree interaction effects on soil microbial communities and functions and are calling for better integration of within‐plot variability to understand biodiversity–ecosystem functioning relationships.

## Graphical abstract

In our study, we were able to show in a subtropical tree diversity experiment (BEF‐China) that soil microbial biomass and respiration show a fine spatial distribution pattern between trees. This suggests that tree–tree interactions are driving soil functioning when zooming to the appropriate scale. Our study highlights the need to focus on fine‐scale variability in future research.

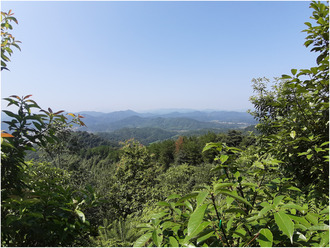

## 1 INTRODUCTION

Forest ecosystems are crucial for the planet's health and sustainability by supporting an extensive range of biodiversity and ecosystem services, including carbon storage, primary production and water and nutrient cycling (Bardgett & Wardle, 2011; van der Heijden et al., 2008; Wagg et al., 2014). The potential of carbon storage within a forest depends on the interactions with the environment and the dominant management practices (Erb et al., 2013). Especially carbon source–sink dynamics are significantly influenced by the interactions between soil microbes and understory plants (Xu et al., 2020).

Microbial‐driven decomposition of organic matter and nutrient cycling is essential for maintaining ecosystem productivity in many different biomes (Delgado‐Baquerizo et al., 2016; Gottschall et al., 2019; Gougoulias et al., 2014; van der Heijden et al., 2008). Microbes are the primary drivers of belowground carbon storage in forests (Schmidt et al., 2011). They transform organic carbon into stable soil organic matter through processes like aggregation or accumulation of microbial necromass (Buckeridge et al., 2020; Miltner et al., 2012; Wang et al., 2021). Thus, this stabilisation of the forest carbon pool provides tools to mitigate climate change (Bastin et al., 2019; Lewis et al., 2019). Understanding the drivers of belowground carbon storage and its relationship with biodiversity is crucial for effective forest management and carbon sequestration (Messier et al., 2022). In particular, soil microbial biomass and respiration could serve as a proxy for nutrient cycling and soil organic matter turnover (Crowther et al., 2019) and were shown to be correlated with soil carbon sequestration (Beugnon, Bu, et al., 2023; Lange et al., 2015). Therefore, these soil microbial properties together can provide important information on multiple soil ecosystem functions (Eisenhauer et al., 2018).

Microbial properties generally vary between soil layers due to lower resource availability (e.g. nutrients and oxygen) in the deeper soil layers leading to reduced microbial diversity and biomass (Goebes et al., 2019; Jobbágy & Jackson, 2001). However, rhizodeposition can increase microbial activity at deeper soil layers (Lopez et al., 2020), potentially leading to different drivers of microbial activity and biomass across soil layers (Blume et al., 2002; Loeppmann et al., 2016).

Tree diversity was shown to enhance soil microbial diversity, abundance and functioning, leading to improved nutrient cycling, organic matter decomposition and carbon storage (Beugnon et al., 2021; Gamfeldt et al., 2013; Gottschall et al., 2019; Li et al., 2019; Pei et al., 2016); primarily due to higher diversity of substrates from litterfall and rhizodeposition as well as possible increased belowground interactions with tree species‐specific soil microbes (Beugnon, Eisenhauer, et al., 2023; Huang et al., 2017). However, other studies showed that the tree diversity impact on soil microbial functions is non‐significant, varies across functional groups such as bacteria and fungi (Cesarz et al., 2022; Rivest et al., 2019) or is less important than tree identity effects or abiotic conditions (Cesarz et al., 2022; Tedersoo et al., 2016; Yamamura et al., 2013). There are now empirical pieces of evidence that spatio‐temporal dynamics along tree diversity gradients can drive soil microbial functions (Gottschall et al., 2022), which vary with the tree neighbourhood (Trogisch et al., 2021).

Forest soils' spatial structure and processes can become highly heterogeneous due to the spatial distribution of roots and root inputs. Soil respiration, for instance, was shown to be higher at the base of birch trees compared to 150 cm away, indicating ‘hot‐spots’ of soil microbial activity close to the tree (Parker et al., 2017). This spatial distribution of soil functions is crucial when considering interactions between trees or with the understory vegetation (Kuzyakov & Blagodatskaya, 2015; Mao et al., 2015). Microbial communities were found to be more active and diverse when surrounded by neighbouring trees than when close to an isolated tree (Habiyaremye et al., 2020). Especially, the effects of tree–tree interactions are expected to be maximised in the interaction zone between the trees (Trogisch et al., 2021). This highlights the role of the neighbouring trees on the functioning of soil microbes in forest ecosystems, especially in the context of highly diverse forests. However, information on the spatial distribution of soil processes (e.g. soil respiration) at finer spatial scales is missing (Friggens et al., 2020).

In this study, we aimed to understand the effects of tree–tree interactions on soil microbial biomass and respiration and their spatial distribution. We set up small‐scale transects in tree neighbourhoods in a Chinese subtropical forest experiment (BEF‐China), where we tested the following hypotheses: (H1) In mono‐specific tree pairs, we expect decreasing microbial biomass and respiration with increasing distance from the trees and with increasing soil depth, due to lower resource availability in greater distances. (H2) Due to higher complementarity as well as quantity and diversity of resource inputs between hetero‐specific tree pairs, we expect overall higher microbial biomass and respiration than in mono‐specific pairs. (H3) We expect the interaction between hetero‐specific tree pairs to be maximised in the interaction zone between the two trees; thus, soil microbial biomass and respiration are highest in the topsoil in the middle of the transect between two adjacent trees.

## 2 MATERIALS AND METHODS

### 2.1 Study site

The study site was located in south‐east China near the City of Xingangshan, Jiangxi Province (29.12° N, 117.90° E), and is part of the BEF‐China experiment (Bruelheide et al., 2014). The experiment was planted in 2009, after a clear‐cut of the previous commercial plantation of *Pinus massoniana* and *Cunninghamia lanceolata*, and it covers an area of 26.7 ha, ranging in altitude from 105 to 275 m. The region has a subtropical climate, with warm, humid summers and cool, dry winters. The local mean annual temperature is 16.7°C with an annual precipitation of 1821 mm (Yang et al., 2013). The soils of this region are Cambisols and Cambisol derivatives, with Regosols on ridges and crests (Geißler et al., 2012; Scholten et al., 2017). The natural vegetation of the region is characterised by species‐rich, broad‐leaved, subtropical forests dominated by evergreen and deciduous species such as *Castanopsis eyrei*, *Cyclobalanopsis glauca*, *Daphniphyllum oldhamii* and *Lithocarpus glaber* (Bruelheide et al., 2011, 2014).

### 2.2 Study design and field sampling

**FIGURE 1:**
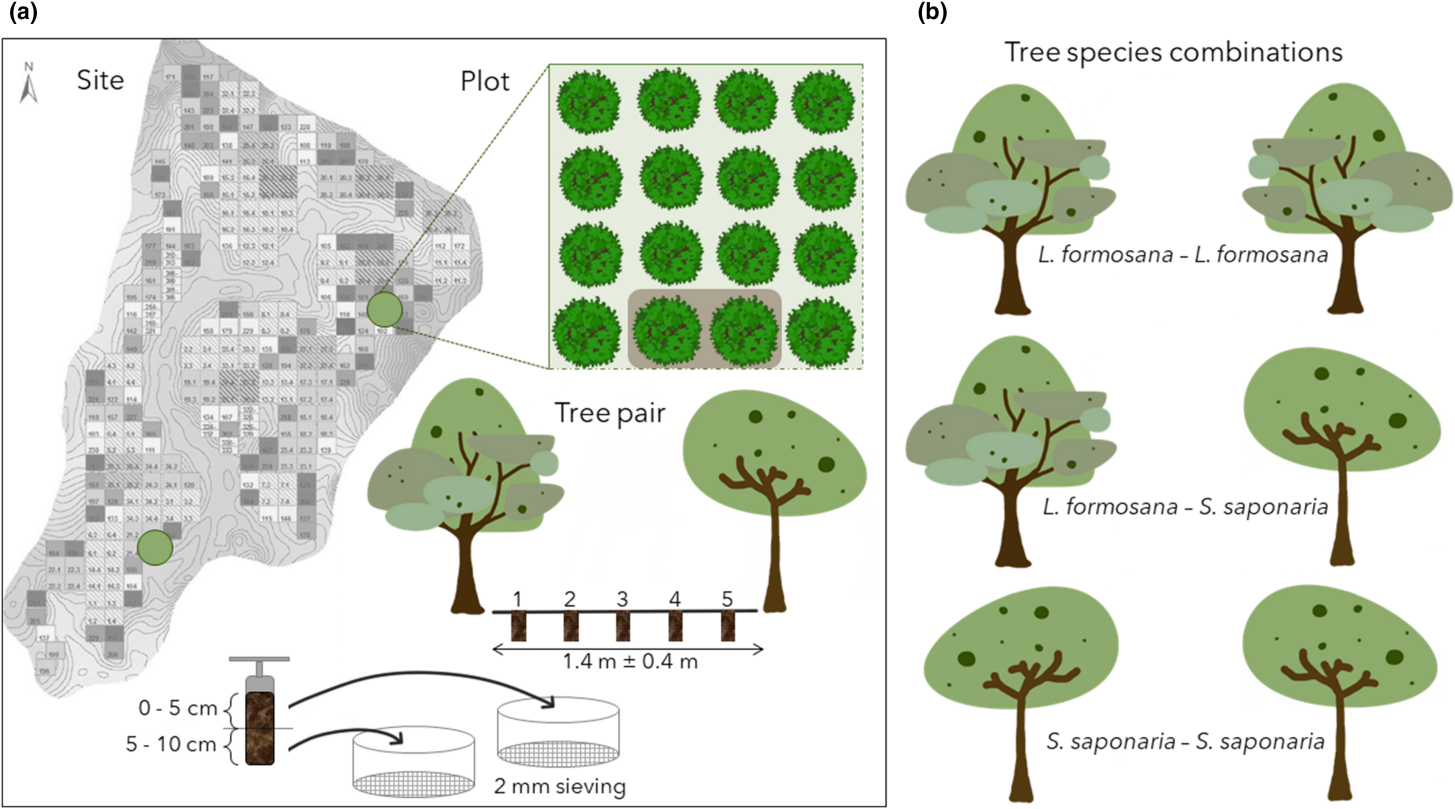
Sampling Design. Overview of the BEF‐China experimental Site a (Panel a) with the two sampled plots (green) representing two‐species mixture plots. Plot tree planting grid pattern with a marked tree pair, here only a small section of the 20 × 20 trees plot was drawn. Positions of the five soil cores between the tree pair with the in‐core division of the depths. Tree species combinations are shown in Panel b.

We selected two plots with the same species mixture of the deciduous tree species *Liquidambar formosana* and *Sapindus saponaria*. The selected tree species have significant and dissimilar effects on soil microbial properties (Beugnon et al., 2021). In each plot, we selected five replicates of both mono‐specific pairs (*L. formosana–L. formosana* and *S. saponaria*–*S. saponaria*) and of the hetero‐specific pair (*L. formosana*–*S. saponaria*). The litter layer was removed prior to sampling. Prior to sampling the soil, the exact distance between the trees was measured to ensure an equal distribution of the sampling positions; the mean distance between the trees was 1.4 ± 0.4 m. To measure the spatial distribution of soil microbial biomass and respiration, we took five soil cores on the transect line between each pair using 5‐cm‐diameter soil cores. To test for the effect of soil depth on soil microbial biomass and respiration, each soil core was split into depths of 0–5 cm and 5–10 cm and sieved through a 2 mm mesh (Figure 1). The soil samples were stored at −20°C until being analysed approximately 2 months later. Altogether, 300 soil samples were collected from two plots, three combinations of trees replicated five times, five positions and two depths. Additionally, the tree diameter at breast height (DBH) was measured for each tree pair to calculate tree biomass, following Beugnon, Bu, et al. (2023).

### 2.3 Soil microbial biomass and respiration measurements

We measured soil microbial biomass (Cmic) and respiration using 6 g of fresh soil on an automated O_2_ micro‐compensation apparatus (Scheu, 1992). Soil microbial respiration was measured first, as the oxygen consumption per hour per dry weight of soil in μl (respiration given in μL O_2_ g^−1^ dry weight h^−1^). This reflects the active part of the soil microbial community at the sampling time. Afterwards, the microbial biomass (given in μg microbial carbon g^−1^ dry weight) was measured by adding glucose (8 mg per gram of dry soil) to the samples (substrate‐induced respiration [SIR] method). The microbial biomass measurement was recorded for approximately 12 h to quantify the total metabolically active biomass of soil microorganisms of the sample (Scheu, 1992).

### 2.4 Statistical analysis

A description of all the variables used in this study can be found in Table S1. All data handling and statistical calculations were performed using the R statistical software version 4.2.2 (www.r‐project.org), and R‐scripts are provided on https://github.com/henriettechristel/Soil‐microbes_Tree‐Interaction.git, model fit and statistical assumptions can be found in Supplement S2.

#### 2.4.1 Spatial distribution of microbial biomass and respiration (H1)

To test the effects of distance to the closest tree and depth on the soil microbial biomass and respiration, we used linear mixed‐effects models and normal distribution assumptions that included plot as a random effect, and distance and depth as fixed effects. The model was fitted on mono‐specific pairs and was used to predict the soil microbial properties over a distance to the closest tree from 0 to 90 cm and a depth from 0 to 10 cm (Supplement S2).

#### 2.4.2 Belowground overyielding between hetero‐specific tree pairs (H2)

Belowground overyielding of soil microbial biomass and respiration was calculated as the difference between observed soil microbial properties between a hetero‐specific pair and what would be expected based on the weighted means of the mono‐specific pairs for a given position between the trees 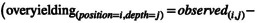 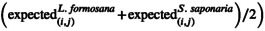, where ‘*i*’ is the position between the trees and ‘*j*’ is the depth. Positive results indicate soil microbial properties in mixed pairs are overyielding (i.e. producing more biomass or respiration than expected based on mono‐specific pairs), and negative results indicate soil microbial properties in mixed pairs are underyielding (i.e. producing less biomass or respiration than expected based on mono‐specific pairs). The expected values were predicted from the model fits from H1.

We used belowground overyielding as a response variable to test for the effect of the hetero‐specific pair on the average value of the soil samples. Additionally, we tested for the effect of depth using a linear mixed‐effects model with plot as random effect and pair as fixed effect.

To determine differences between soil depths, we used a Tukey HSD test based on an analysis of variance (ANOVA type 1).

#### 2.4.3 Spatial distribution of belowground overyielding (H3)

To test the effects of distance to the tree species and depth on the belowground overyielding of soil microbial biomass and respiration, we used linear mixed‐effects models, which included plot as a random effect and distance in centimetres from the trees and depth as fixed effects (Supplement S2). We fixed the positions of the trees to *L. formosana* being tree 1 and *S. saponaria* being tree 2 in a mixed pair. Like this, the positioning of the trees was fixed within the data and could be analysed in terms of a spatial gradient.

All linear mixed‐effect models were fitted, using the ‘lmer’ function of the R package *lme4* (Bates et al., 2015). To define the quality of the model fits of all used linear mixed‐effects models, the ‘check_model’ function of the R package *performance* (Lüdecke et al., 2021) was used to investigate various model assumptions, such as normality of residuals, normality of random effects, linear relationship, homogeneity of variance and multicollinearity (Briggs & Cheek, 1986).

## 3 RESULTS

The analyses showed on average a high variability in soil microbial biomass (mean ± SD = 381.48 ± 137.02 μg Cmic g^−1^ dry weight) and soil basal respiration (1.77 ± 0.93 μL O_2_ g^−1^ dry weight h^−1^) among the samples from the investigated plots.

### 3.1 Spatial distribution of soil microbial biomass and respiration (H1)

The analyses showed on average higher microbial biomass (mean ± SD = 380.97 + 123.49 μg Cmic g^−1^ dry weight) and respiration (1.91 + 0.93 μL O_2_ g^−1^ dry weight h^−1^) in the mono‐specific tree pairs of *Sapindus saponaria* in comparison to *Liquidambar formosana* (microbial biomass: mean ± SD = 355.05 ± 138.55 μg Cmic g^−1^ dry weight; microbial respiration: 1.65 ± 0.77 μL O_2_ g^−1^ dry weight h^−1^). However, we could observe a similar trend in relation to space for both species (Figures S2 and S3).

**FIGURE 2:**
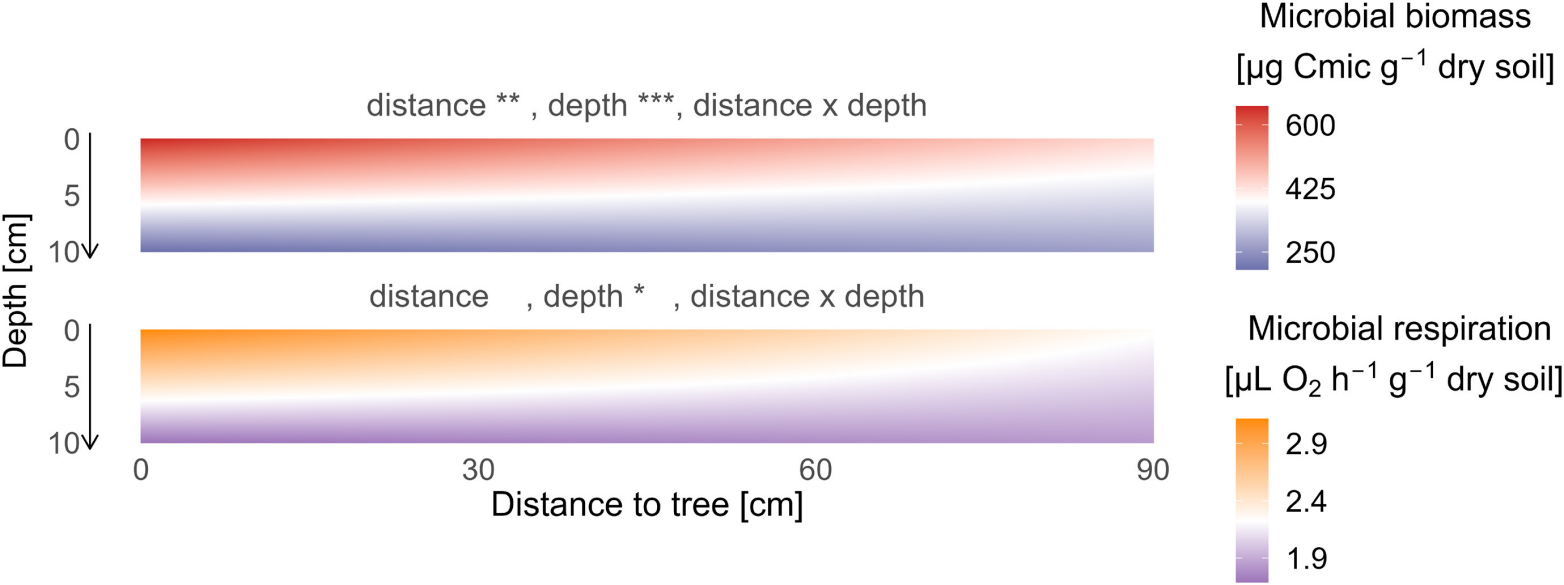
Distance to tree and depth effects on microbial biomass (top) and respiration (bottom) in mono‐specific tree pairs. Effects are predicted from the model (*soil properties* ~ *depth* × *distance to tree*) with plot as random effect. Distance to tree reports the distance to the closest tree from mono‐specific tree pairs. Microbial biomass is coloured blue (low) to red (high), and microbial respiration is coloured purple (low) to orange (high). The significance levels were standardised across the panels (*p < .05; **p < .01; ***p < .001).

Soil microbial biomass decreased significantly with increasing distance to the tree (estimate ± SE = −1.60 ± 0.57 μg Cmic g^−1^ dry weight cm^−1^, *p* = .006) and with soil depth (−44.96 ± 8.20 μg Cmic g^−1^ dry weight cm^−1^, *p* < .001, Figure 2). The interaction of distance and depth was not significant (*p* = .064). Likewise, soil microbial respiration decreased with increasing depth (−0.14 ± 0.06 μL O_2_ g^−1^ dry weight h^−1^ cm^−1^, *p* = .01), but distance to the tree and the interaction of distance and depth had no significant effects (*p* = .08, *p* = .315, respectively, Figure 2).

### 3.2 Spatial distribution of belowground overyielding (H2–H3)

**FIGURE 3:**
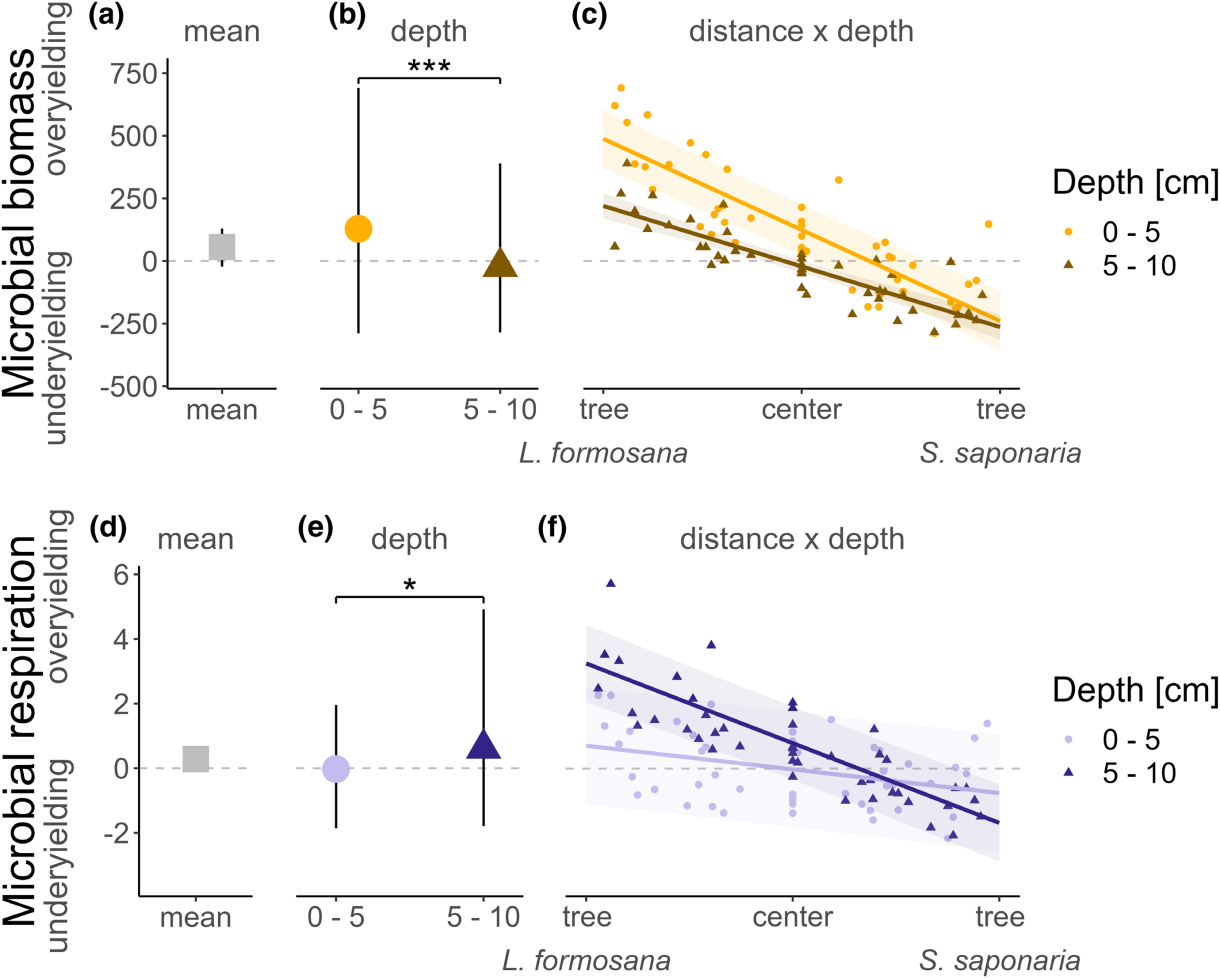
Distance to tree and depth effects on microbial biomass (top) and respiration (bottom) overyielding in hetero‐specific tree pairs. The mean value (grey square) of overyielding for microbial biomass (a) and respiration (d) across all depths and positions, for each depth (microbial biomass: b, yellow circle for 0–5 cm; brown triangle for 5–10 cm, and microbial respiration: e, light blue circle for 0–5 cm; dark blue triangle for 5–10 cm) and each sampling point by depth (microbial biomass: C and respiration: f). Confidence intervals were predicted from models using the ‘ggpredict’ function of the R package ggeffects. The significance levels were standardised across the panels (*p < .05; ***p < .001).

Our analyses showed no general belowground overyielding for microbial biomass or respiration between the mixed tree species pairs (Figure 3a,d). An increasing depth reduced soil microbial biomass overyielding (estimate ± SE = −150.67 ± 42.17 μg Cmic g^−1^ dry weight cm^−1^, *p* < .001) but increased microbial respiration overyielding (0.71 ± 0.21 μL O_2_ g^−1^ dry weight h^−1^ cm^−1^, *p* = .001, Figure 3b,e, respectively).

We studied the spatial distribution of microbial properties overyielding by testing for the interacting effects of increasing distance to *L. formosana* (tree 1, i.e. closeness to tree 2) and depth.

We found that an increased distance (from *L. formosana*) had a significant negative effect on the microbial biomass overyielding (−452.51 ± 34.90 μg Cmic g^−1^ dry weight cm^−1^, *p* < .001). The analysis showed overyielding close to *L. formosana* (i.e. tree 1), turning into underyielding close to *S. saponaria* (i.e. tree 2, Figure 3c). This pattern was even stronger in the shallower soil (0–5 cm) compared to the deeper soil layer (5–10 cm), as indicated by a significant interaction effect between distance and depth (interaction estimate ± SE = 30.01 ± 10.07 μg Cmic g^−1^ dry weight cm^−1^, *p* = .004). We found similar results for the overyielding of microbial respiration: increased distance to *L. formosana* (tree 1) decreased the microbial respiration overyielding significantly (−0.77 ± 0.19 μL O_2_ g^−1^ dry weight h^−1^ cm^−1^, *p* < .001) with overyielding close to *L. formosana*, and underyielding close to *S. saponaria* (Figure 3f). Contrary to microbial biomass, this pattern was stronger in the deeper soil compared to the shallower soil layer (interaction estimate ± SE = −0.38 ± 0.05 μg Cmic g^−1^ dry weight cm^−1^, *p* < .001).

## 4 DISCUSSION

In this study, we tested the effect of tree–tree interactions and the spatial distribution of microbial biomass and respiration in subtropical forest soils. We found negative effects of increased distance to the tree and depth on soil microbial biomass and respiration within mono‐specific pairs. Although the effects were different for *Liquidambar formosana* and *Sapindus saponaria*, we observed similar trends in relation to space. In the hetero‐specific pairs, we did not find any significant tree–tree interaction effects on the average soil sample value at the species pair level. However, there were substantial variations in tree–tree interaction effects at the small spatial scale. In fact, effects on both soil microbial properties were spatially distributed, ranging from overyielding near *Liquidambar formosana* to underyielding close to *Sapindus saponaria*.

### 4.1 Spatial distribution of soil microbial biomass and respiration (H1)

Soil microbial biomass decreased with increasing distance from the trees in mono‐specific tree pairs. This could be explained on the one hand by higher water availability due to stemflow near the tree base, which can leach and transport nutrients and microorganisms from the canopy layer to the soils (Bittar et al., 2018). Soil moisture was shown to be important in many studies before (Cesarz et al., 2022; Schimel, 2018; Serna‐Chavez et al., 2013); we also found this effect in our study (see Figure S6). High levels of soil moisture can increase soil enzyme activities, fluxes of soil nutrients and oxygen availability (Brockett et al., 2012; Stark & Firestone, 1995), and higher soil humidity can furthermore buffer possible negative changes in soil pH, suggesting it to be a key driver of soil microbial biomass (Cesarz et al., 2022). On the other hand it could also be explained by a higher rhizodeposition closer to the trees (Parker et al., 2017). Our findings would suggest the importance of forest density in modulating soil functioning.

As expected, we also found a negative effect of soil depth on both microbial properties. This is in line with previous findings where a lower amount of carbon and nutrients was found in deeper soil layers, as the main decomposition happens in the leaf litter cover and top soil layers (Goebes et al., 2019; Jobbágy & Jackson, 2001; Prescott & Grayston, 2013). Additionally, deeper soil layers often have a decreased amount of oxygen, soil water content and contain less plant root biomass (Engelhardt et al., 2018; Fall et al., 2012; Serna‐Chavez et al., 2013). Thus, the present results at the small scale are in line with previous findings at the larger scales, where soil organic carbon decreased with increasing soil depth and distance to trees (Rabearison et al., 2023). These similar results suggest that understanding interaction effects at small scales have the potential to be upscaled.

### 4.2 Spatial distribution of belowground overyielding (H2–H3)

Our study showed no overyielding for the average value (across all soil core positions and depth layers). However, we found significant differences between the soil depths for both soil microbial functions. Microbial biomass showed, on average, higher overyielding in the shallower soil (0–5 cm), whereas microbial respiration showed higher overyielding in the deeper soil (5–10 cm). The BEF‐China experimental Site A was established in 2009 after a clear‐cut of the previous plantation (Yang et al., 2013). In grasslands, plant diversity effects on soil organic matter are getting stronger in the topsoil layer over time (Lange et al., 2023). These findings from experimental grasslands are also suggested in forests top soil layers since soil microbial biomass and organic matter content are affected by forest productivity (Beugnon, Eisenhauer, et al., 2023), and tree diversity effects on productivity could get stronger with stand age (Huang et al., 2018; Perles‐Garcia et al., 2021). This could indicate that overyielding would similarly increase over time in diverse stands. The increased average overyielding of microbial respiration in the deeper soils could suggest increased carbon sequestration by adding soil microbial necromass to the carbon pool. Higher microbial respiration indicates a higher microbial activity related to the decomposition and recycling of fresh material. Here, easily accessible organic matter such as leaves are turned into more stable forms of organic matter, for example, microbial necromass, and contributes to carbon storage over time (Buckeridge et al., 2020; Schmidt et al., 2011). A higher respiration can indicate a higher release of carbon dioxide into the atmosphere. However, it was also shown that higher plant diversity and therefore increased rhizosphere carbon input can result in both increased microbial activity and carbon storage (Lange et al., 2015).

We expected hetero‐specific tree–tree interaction effects to be maximised in the middle between the planted trees. Contrary to our hypothesis (H3), we found an overyielding of soil microbial biomass and respiration close to *L. formosana* and underyielding close to *S. saponaria*, showing that microbes in mixed pairs perform better than expected close to *L. formosana* but less well than expected close to *S. saponaria*. The gradient from over‐ to underyielding of microbial respiration was less pronounced in the shallower soil than in the deeper soil. This could indicate that the presence of *S. saponaria* had a positive effect on microbes close to *L. formosana* and it is stronger in the deeper soil layer (5–10 cm), possibly through fine root exudates (Zheng et al., 2017). Microbial respiration was less affected by the mixture than microbial biomass in the topsoil layer (0–5 cm). It was shown that the balsam of *L. formosana* contains acidic compounds, which were reported to be inhibitory for fungi (Chien et al., 2013). These could also be present in the leaf litter or root exudates (Öztürk et al., 2008) and inhibit microbial respiration more than microbial biomass. Together with a spatial distribution of litter in the hetero‐specific pairs (Beugnon, Eisenhauer, et al., 2023), this might lead to a small‐scale change in soil pH. Soil pH was found to be a strong driver of microbial growth (Fierer & Jackson, 2006), and additional pH measurements should be performed in future studies to better understand the opposing species identity effects of *S. saponaria* and *L. formosana*. It was shown that soil fungi and bacteria react differently to changes in soil pH: bacterial growth decreased with a more acidic pH, whereas fungal growth was shown to increase (Rousk et al., 2009). This might also explain the significant negative effect of *L. formosana* on microbial respiration in this experiment (Supplement S2: Figure S6). To better understand distribution patterns of microbial properties, belowground tree traits (e.g. specific root length and root diameter) should be taken into account. Recent studies could link them to carbon exudation and fine root density (Bergmann et al., 2020; Sun et al., 2021), as well as soil organic matter decomposition (Adamczyk et al., 2019).

The positive tree–tree interaction effect of the hetero‐specific tree pair on soil microbial biomass and respiration shows that neighbourhood effects are acting at small spatial scales, which could explain the inconsistencies of BEF relationships reported in previous forest studies (Beugnon et al., 2021; Cesarz et al., 2022; Li et al., 2019; Pei et al., 2016). Our results stress the need to standardise sampling methods by considering small‐scale interactions to understand the mechanisms behind tree–soil interactions. In addition, measurements of soil microbial properties across a wider range of species transects are now needed to better understand tree–tree interactions in space and their biological drivers.

## 5 CONCLUSION

In the present study, we were able to show in a subtropical tree diversity experiment (BEF‐China) that soil microbial biomass and respiration show a fine spatial pattern in the tree‐tree interaction zone, both vertically and horizontally. Whereas the average value of the soil samples was not affected by tree–tree interactions, tree–tree interactions ranged from overyielding close to *Liquidambar formosana* to underyielding close to *Sapindus saponaria*. Our findings suggest that tree–tree interactions are driving soil functioning when zooming to the appropriate spatial scale. Therefore, in order to understand relationships between trees and soil processes, future research should focus on fine‐scale spatial variability (Eisenhauer et al., 2023).

## AUTHOR CONTRIBUTIONS

**Henriette Christel:** Conceptualization (equal); data curation (lead); formal analysis (lead); investigation (lead); visualization (equal); writing – original draft (lead); writing – review and editing (equal). **Helge Bruelheide:** Writing – review and editing (supporting). **Simone Cesarz:** Conceptualization (equal); funding acquisition (equal); supervision (supporting); writing – review and editing (supporting). **Nico Eisenhauer:** Conceptualization (equal); funding acquisition (equal); supervision (supporting); writing – review and editing (supporting). **Georg J. A. Hähn:** Data curation (supporting); formal analysis (equal); visualization (equal); writing – review and editing (supporting). **Rémy Beugnon:** Conceptualization (equal); formal analysis (supporting); investigation (equal); supervision (lead); visualization (supporting); writing – review and editing (equal).

## CONFLICT OF INTEREST STATEMENT

The authors declare no competing interests.

## Supporting information


Data S1.

## Data Availability

All data and code are published through a public repository (https://github.com/henriettechristel/Soil‐microbes_Tree‐Interaction.git).
