# Supplementary material for: The spatial distribution of tree–tree interaction effects on soil microbial biomass and respiration
Source: Ecol Evol. 2024 Jun 18;14(6):e11530. doi: 10.1002/ece3.11530 (PMC11183910; doi:10.1002/ece3.11530)
Supplement: Supplementary file 1 — Data S1. [file ECE3-14-e11530-s001.zip › Supplementary_1.pdf]

Supplementary Material 1

The spatial distribution of tree-tree interaction effects on soil microbial biomass and respiration

Henriette Christel, Helge Bruelheide, Simone Cesarz, Nico Eisenhauer, Georg J. A. Hähn and Rémy Beugnon

Table S1: Description of variables

Variables	Code	Unit	Calculation
	Tree variables		
**Plot**	plot	none	Treatment
**Diameter at breast height**	dbh	m	Measured
**Basal Area**	ba	m^2	$ba = \frac{(dbh)^2}{4\pi}$
**Distance to closest tree**	close_distance	cm	Measured
**Diameter of closest tree**	close_dbh	cm	Measured
**Biomass of closest tree**	close_biomass	kg_{carbon}	Estimated from ba
**Closest tree species**	close_species	none	Measured
**Distance to tree 1**	distance_T_1	cm	Measured
**Distance to tree 2**	distance_T_2	cm	Measured
**Distance to each tree with soil core 3 as the center point**	dist.T1.center	cm	Measured
**Distance to closest L. formosana**	dist.liqu	cm	Measured
**Distance to closest S. mukorossi**	dist.sapi	cm	Measured
**Tree species combination of the tree pair**	species_mix	none	Measured
	Soil microbial community		
**Soil microbial biomass**	cmic	µg $C_{microbial}$ g^{-1} dry weight	Measured
**Soil microbial biomass overyielding**	cmic_over		Calculated from observed cmic
**Soil microbial respiration**	bas_res	µl O_2 g^{-1} dry weight h^{-1}	Measured
**Soil microbial respiration overyielding**	bas_over		Calculated from observed bas_res
	Environmental variables		
**Soil depth**	depth	cm	Measured
**Root biomass**	root_biomass	g m^{-3}	Measured from soil cores
**Distance to closest understory plant**	plant_distance	cm	Measured
**Soil water content**	rh	%	Measured
