# Supplementary material for: The spatial distribution of tree–tree interaction effects on soil microbial biomass and respiration
Source: Ecol Evol. 2024 Jun 18;14(6):e11530. doi: 10.1002/ece3.11530 (PMC11183910; doi:10.1002/ece3.11530)
Supplement: Supplementary file 1 — Data S1. [file ECE3-14-e11530-s001.zip › Supplementary_2.pdf]

Supplementary Material 2

The spatial distribution of tree-tree interaction effects on soil microbial biomass and respiration

Henriette Christel, Helge, Bruelheide, Simone Cesarz, Nico Eisenhauer, Georg J. A. Hähn and Rémy Beugnon

Contents

Figure S1: Correlation matrix 2
Section S2: Microbial respiration (H1) 3
Section S3: Microbial biomass (H1) 4
Figure S2: Distance and depth effects for *Liquidambar formosana* in monospecific pairs 5
Figure S3: Distance and depth effects for *Sapindus saponaria* in monospecific pairs 6
Figure S4: Distance and depth effects for hetero-specific pairs 7
Section S4: Overyielding microbial respiration (H2) 8
Section S5: Overyielding microbial biomass (H2) 10
Section S6: Spatial distribution of microbial respiration overyielding (H3) 12
Section S7: Spatial distribution of microbial biomass overyielding (H3) 13
Figure S5: Spatial distribution of the absolute values of microbial biomass and respiration 14
Figure S6: Abiotic and biotic drivers of soil microbial biomass and respiration 15

Figure S1: Correlation matrix

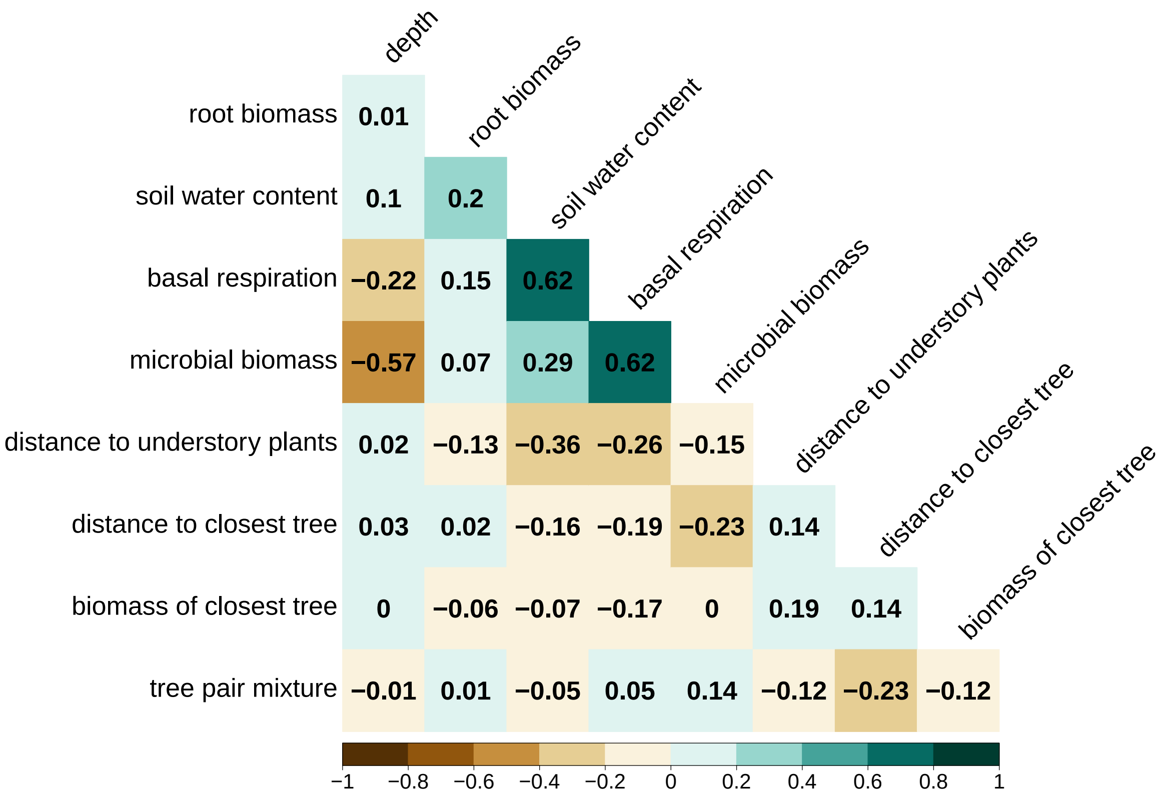

Figure S1: A correlation matrix of the variables used. Brown colour indicates negative correlation, green colour indicates positive correlation.

Section S2: Microbial respiration (H1)

Model summary

lmer(formula = bas_res ~ close_distance * depth + (1|plot), data = df)

	Estimate	Std. Error	t-value	p-value
(Intercept)	2.3262195	0.4764510	4.882389	0.0767479
close_distance	-0.0068739	0.0039270	-1.750412	0.0817317
depth	-0.1427127	0.0563661	-2.531891	0.0121907
close_distance:depth	0.0011358	0.0011281	1.006858	0.3153398

Model fit

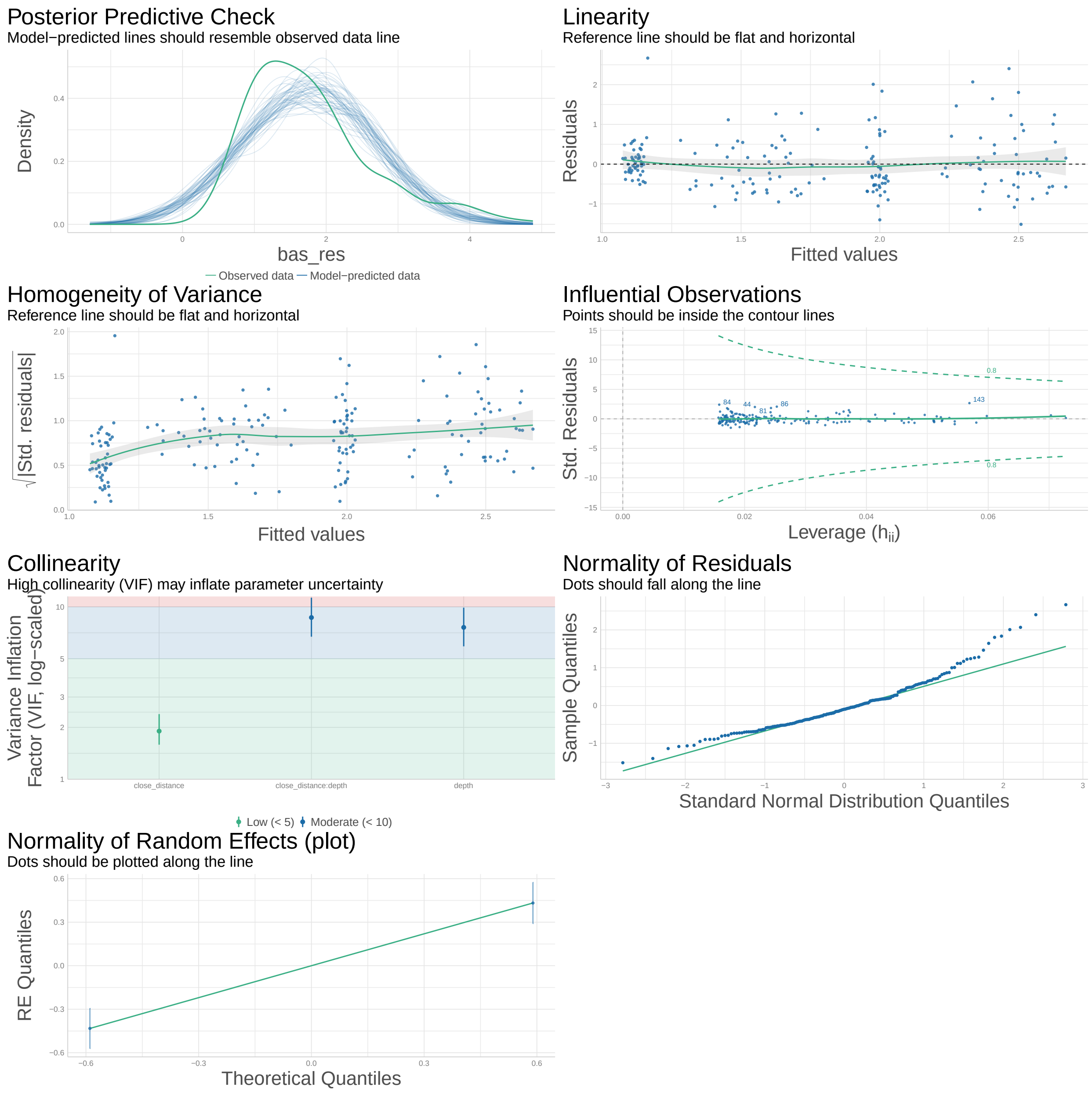

Section S3: Microbial biomass (H1)

Model summary

lmer(formula = cmic ~ close_distance * depth + (1|plot), data = df)

	Estimate	Std. Error	t-value	p-value
(Intercept)	515.8788176	36.9323184	13.968222	0.0000562
close_distance	-1.5954273	0.5709665	-2.794257	0.0057580
depth	-44.9601154	8.1995370	-5.483250	0.0000001
close_distance:depth	0.3061849	0.1640985	1.865861	0.0636699

Model fit

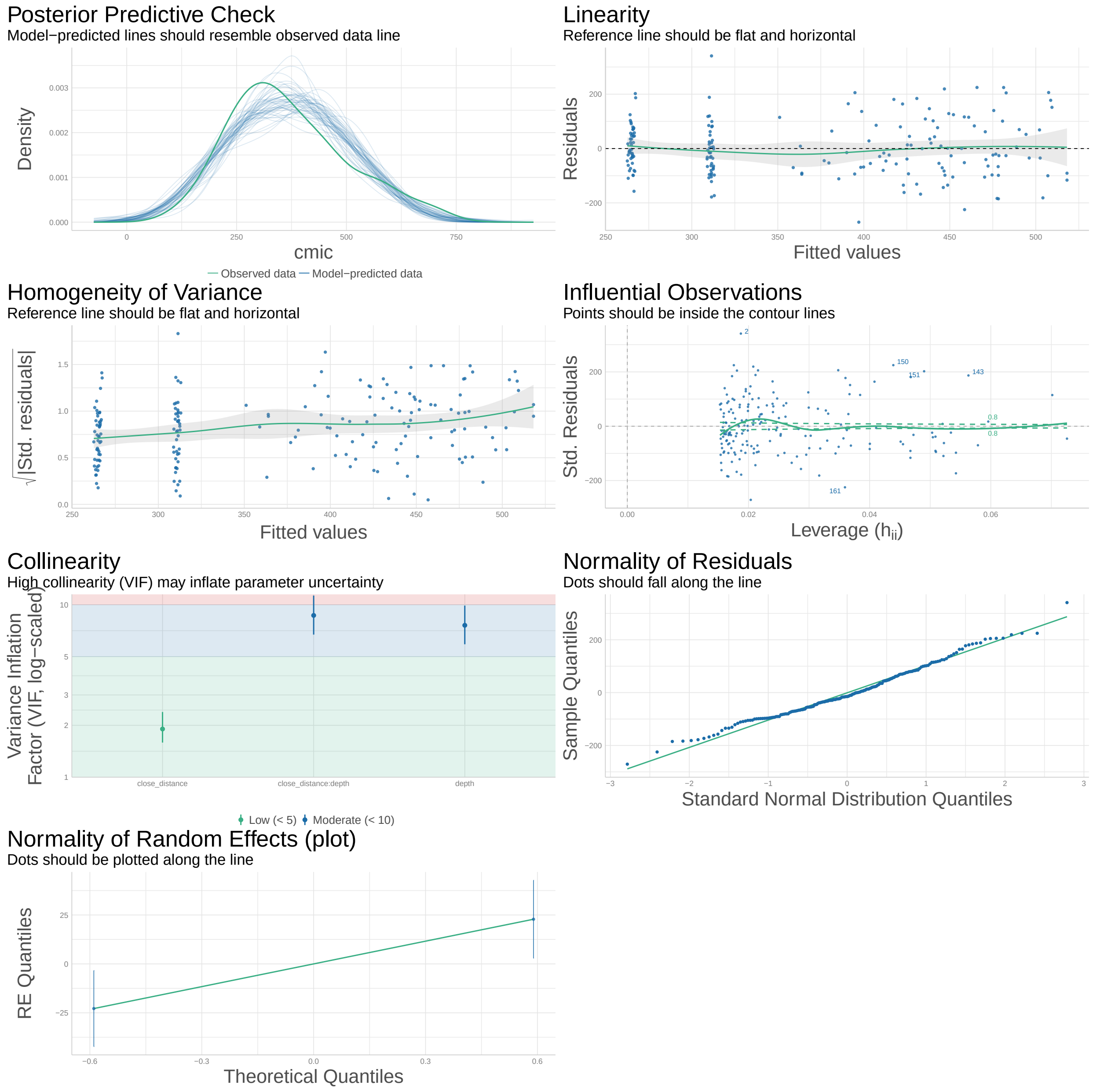

Figure S2: Distance and depth effects for *Liquidambar formosana* in monospecific pairs

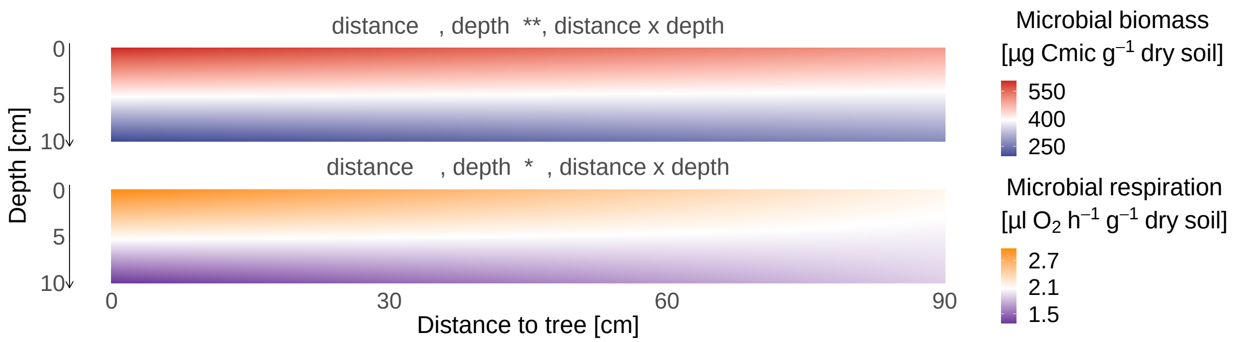

Figure S2: Distance to tree and depth effects on microbial biomass (top) and respiration (bottom) for *Liquidambar formosana* in monospecific tree pairs. Effects are predicted from the model (*soil properties* ~ *depth* * *distance to tree*) with plot as random effect. Distance to tree reports the distance to the closest tree within the monospecific pair. Microbial biomass coloured blue (low) to purple (high), microbial respiration coloured purple (low) to orange (high). The significance levels were standardised across the panels ($p<0.05$: * , $p<0.01$: **, p<0.001:** *).

Figure S3: Distance and depth effects for *Sapindus saponaria* in monospecific pairs

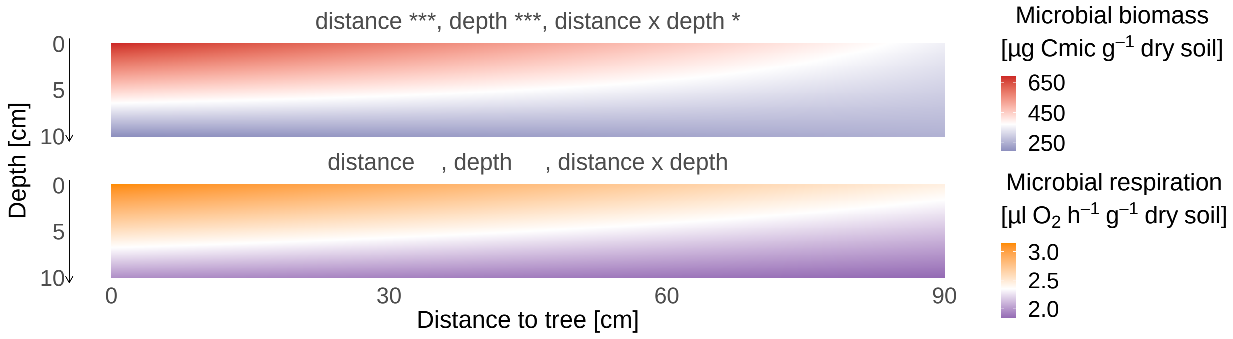

Figure S3: Distance to tree and depth effects on microbial biomass (top) and respiration (bottom) for *Sapindus saponaria* in monospecific tree pairs. Effects are predicted from the model (*soil properties* ~ *depth* * *distance to tree*) with plot as random effect. Distance to tree reports the distance to the closest tree within the monospecific pair. Microbial biomass coloured blue (low) to purple (high), microbial respiration coloured purple (low) to orange (high). The significance levels were standardised across the panels (p<0.05: * , p<0.01: **, p<0.001:** *).

Figure S4: Distance and depth effects for hetero-specific pairs

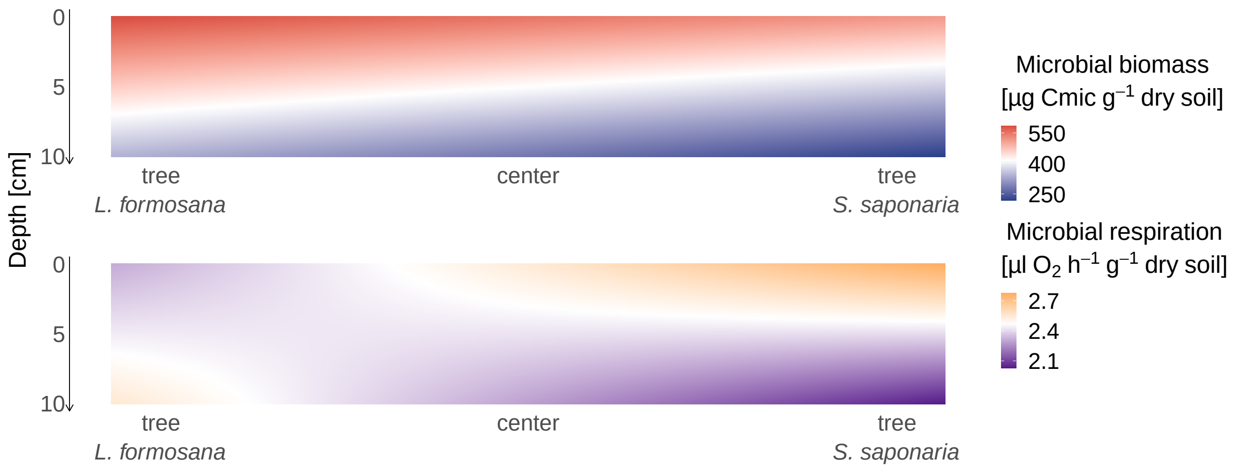

Figure S4: Distance to tree and depth effects on microbial biomass (top) and respiration (bottom) in heterospecific tree pairs. Effects are predicted from the model (*soil properties* ∼ *depth* * *distance to tree*) with plot as random effect. Microbial biomass coloured blue (low) to red (high), microbial respiration coloured purple (low) to high (orange). The significance levels were standardised across the panels ($p < 0.05$: * , $p < 0.01$: ** and $p < 0.001$: ***).

Section S4: Overyielding microbial respiration (H2)

Model summary

lmer(bas_over ~ depth + (1|plot), data = df)

	Estimate	Std. Error	t-value	p-value
(Intercept)	-0.0142504	0.6264277	-0.0227487	0.9853692
depth	0.1416237	0.0428361	3.3061788	0.0013700

Figure 3 Tukey test - depth

	Sum Sq.	Mean Sq.	f-value	p-value
depth %>% as.factor	9.283579	9.283579	6.564345	0.0120856
Residuals	125.867631	1.414243	NA	NA

	Diff	Lower	Upper	p-value
5-0	0.6391505	0.1434713	1.13483	0.0120856

Model fit

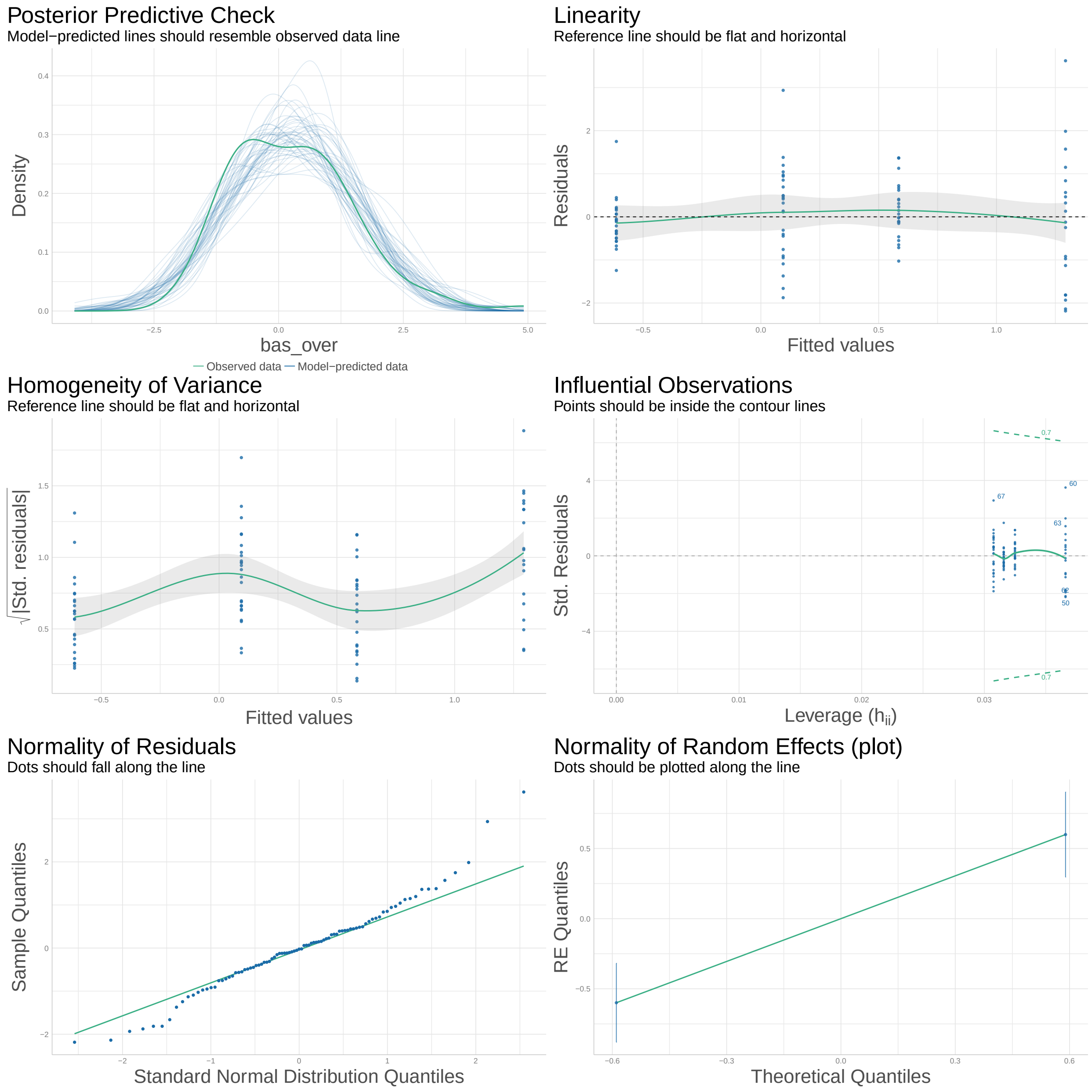
Posterior Predictive Check
Model−predicted lines should resemble observed data line
Density
bas_over
Observed data
Model−predicted data
Linearity
Reference line should be flat and horizontal
Residuals
Fitted values
Homogeneity of Variance
Reference line should be flat and horizontal
√|Std. residuals|
Fitted values
Influential Observations
Points should be inside the contour lines
Std. Residuals
Leverage (h_{ii})
Normality of Residuals
Dots should fall along the line
Sample Quantiles
Standard Normal Distribution Quantiles
Normality of Random Effects (plot)
Dots should be plotted along the line
RE Quantiles
Theoretical Quantiles

Section S5: Overyielding microbial biomass (H2)

Model summary

lmer(cmic_over ~ depth + (1|plot), data = df)

	Estimate	Std. Error	t-value	p-value
(Intercept)	128.57173	29.616232	4.341259	0.0161693
depth	-30.13394	8.433534	-3.573110	0.0005736

Figure 3 Tukey test - depth

	Sum Sq.	Mean Sq.	f-value	p-value
depth %>% as.factor	516533.2	516533.20	12.77892	0.000569
Residuals	3597445.0	40420.73	NA	NA

	Diff	Lower	Upper	p-value
5-0	-150.7629	-234.5623	-66.96356	0.000569

Model fit

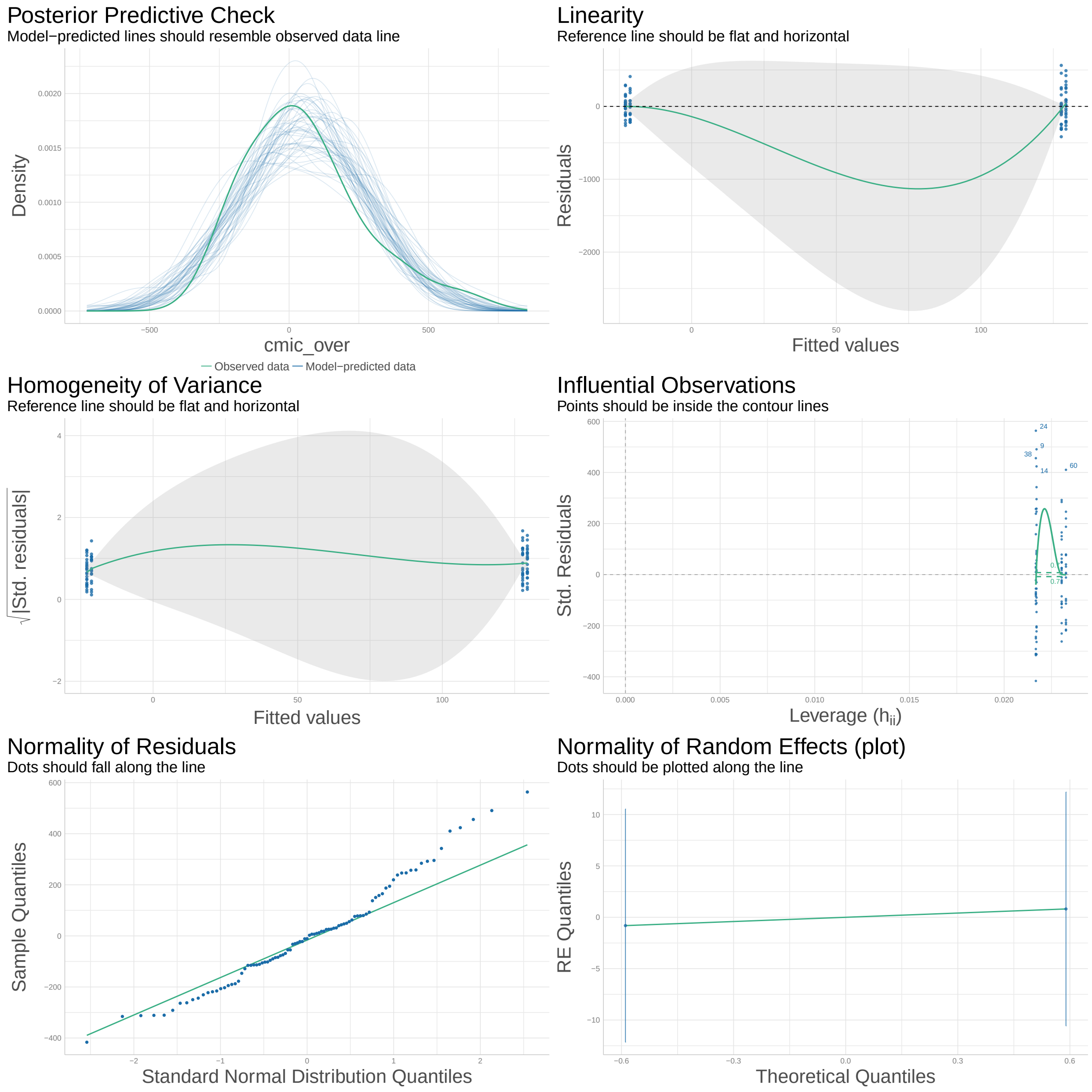
Posterior Predictive Check
Model−predicted lines should resemble observed data line
Density
cmic_over
Observed data — Model−predicted data
Linearity
Reference line should be flat and horizontal
Residuals
Fitted values
Homogeneity of Variance
Reference line should be flat and horizontal
√|Std. residuals|
Fitted values
Influential Observations
Points should be inside the contour lines
Std. Residuals
Leverage (h_{ii})
Normality of Residuals
Dots should fall along the line
Sample Quantiles
Standard Normal Distribution Quantiles
Normality of Random Effects (plot)
Dots should be plotted along the line
RE Quantiles
Theoretical Quantiles

Section S6: Spatial distribution of microbial respiration overyielding (H3)

Model summary

lmer(bas_over ~ dist.T1.center * depth + (1|plot), data = df)

	Estimate	Std. Error	t-value	p-value
(Intercept)	-0.0229950	0.6296411	-0.0365208	0.9766871
dist.T1.center	-0.7741122	0.1876610	-4.1250569	0.0000853
depth	0.1445341	0.0236760	6.1046629	0.0000000
dist.T1.center:depth	-0.3759578	0.0541292	-6.9455595	0.0000000

Model fit

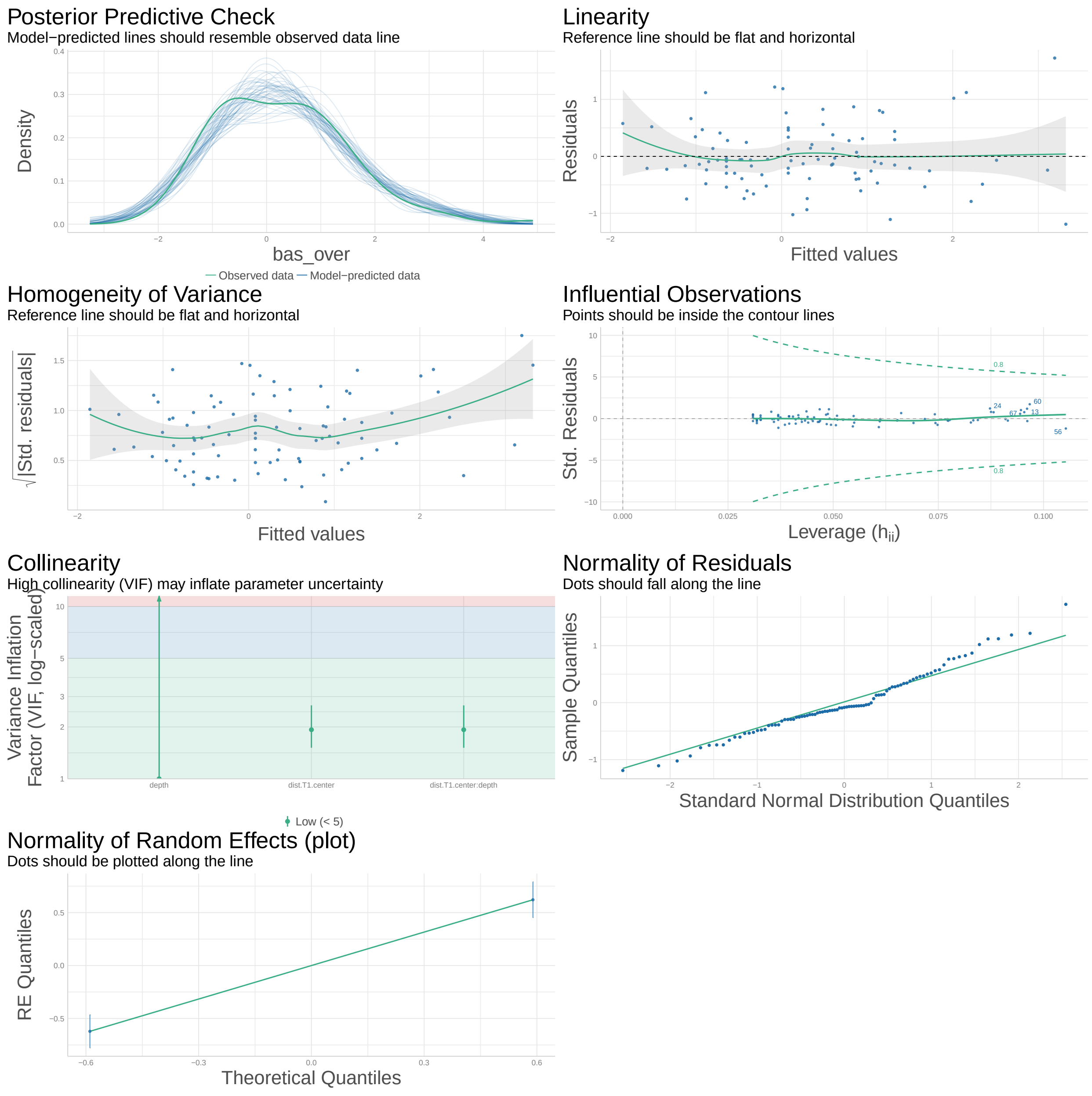

Section S7: Spatial distribution of microbial biomass overyielding (H3)

Model summary

lmer(cmic_over ~ dist.T1.center * depth + (1|plot), data = df)

	Estimate	Std. Error	t-value	p-value
(Intercept)	123.62549	28.254908	4.375363	0.0930928
dist.T1.center	-452.50479	34.902000	-12.965010	0.0000000
depth	-28.51567	4.402464	-6.477206	0.0000000
dist.T1.center:depth	30.01283	10.067663	2.981112	0.0037344

Model fit

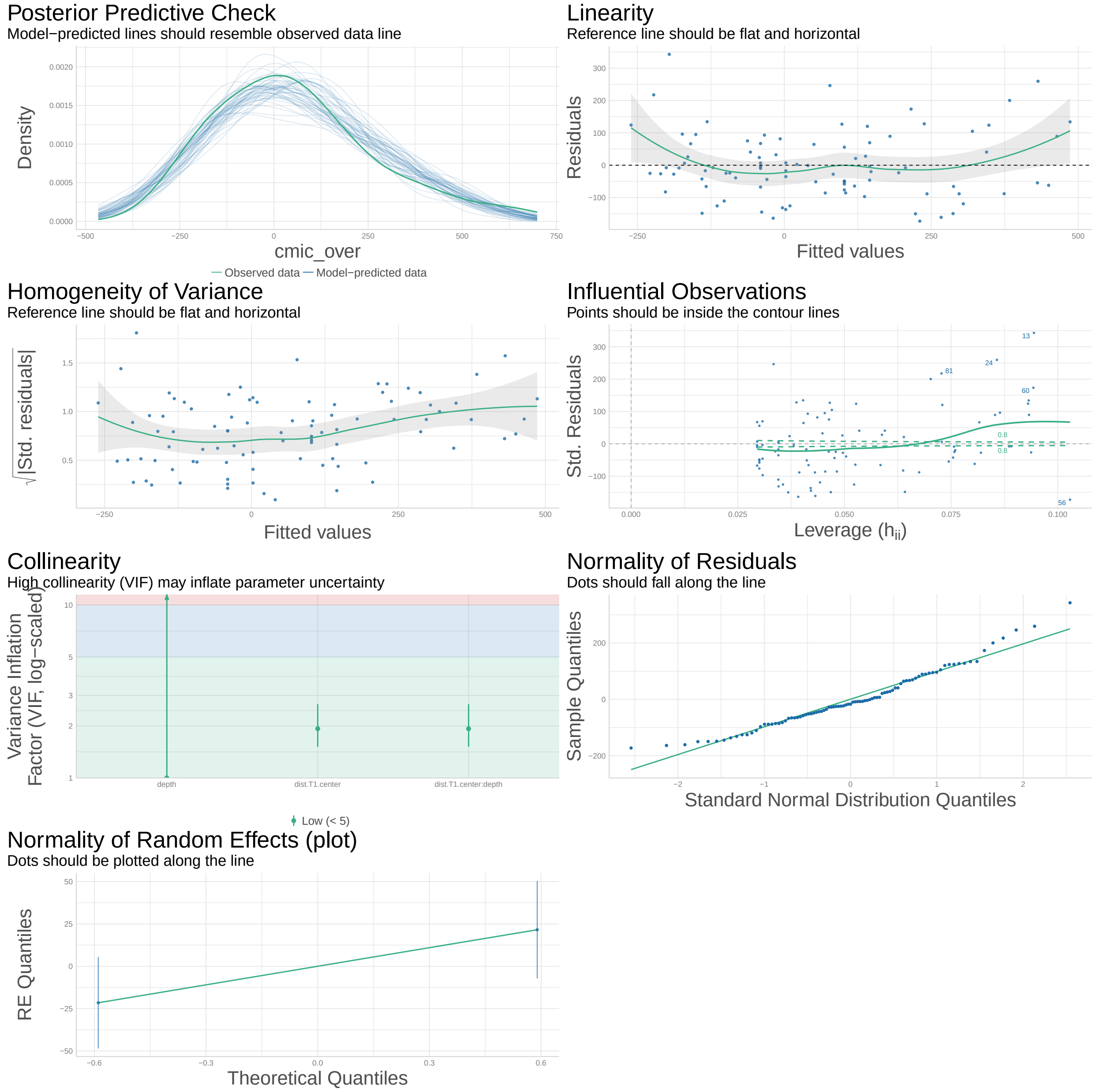

Figure S5: Spatial distribution of the absolute values of microbial biomass and respiration

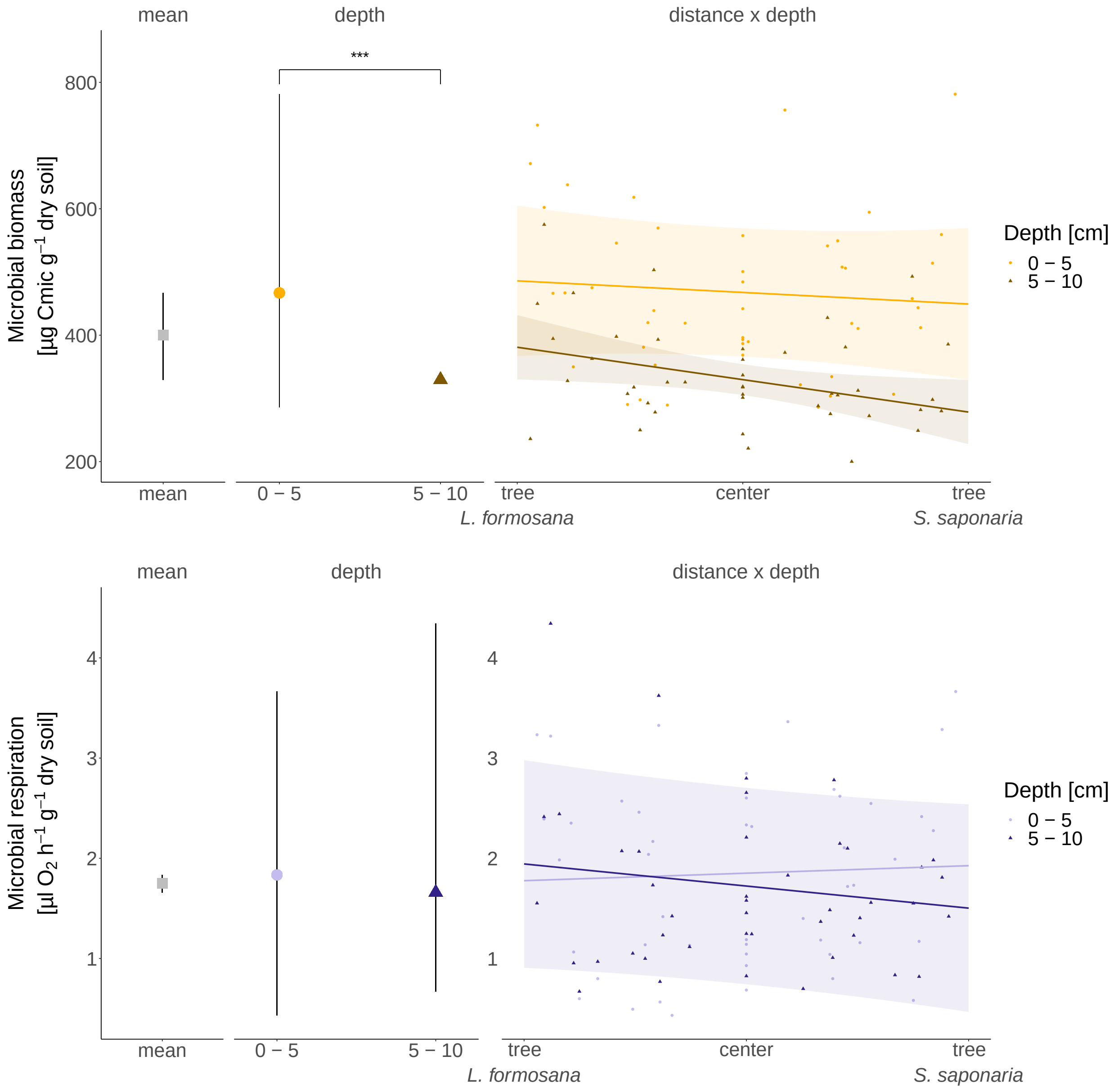

Figure S5: Distance to tree and depth effects on microbial biomass (top) and respiration (bottom) in heterospecific tree pairs. The mean value (grey square) of microbial biomass (A) and respiration (D) across all depths and positions, for each depth (microbial biomass: B, yellow circle for 0-5 cm; brown triangle for 5-10 cm, and microbial respiration: E, light blue circle for 0-5 cm; dark blue triangle for 5-10 cm), and for each sampling point by depth (microbial biomass: C and respiration: F). Confidence intervals were predicted from models using the “ggpredict” function of the R package *ggeffects*. The significance levels were standardised across the panels ($p < 0.05$: * , $p < 0.01$: ** and $p < 0.001$: ***).

Figure S6: Abiotic and biotic drivers of soil microbial biomass and respiration

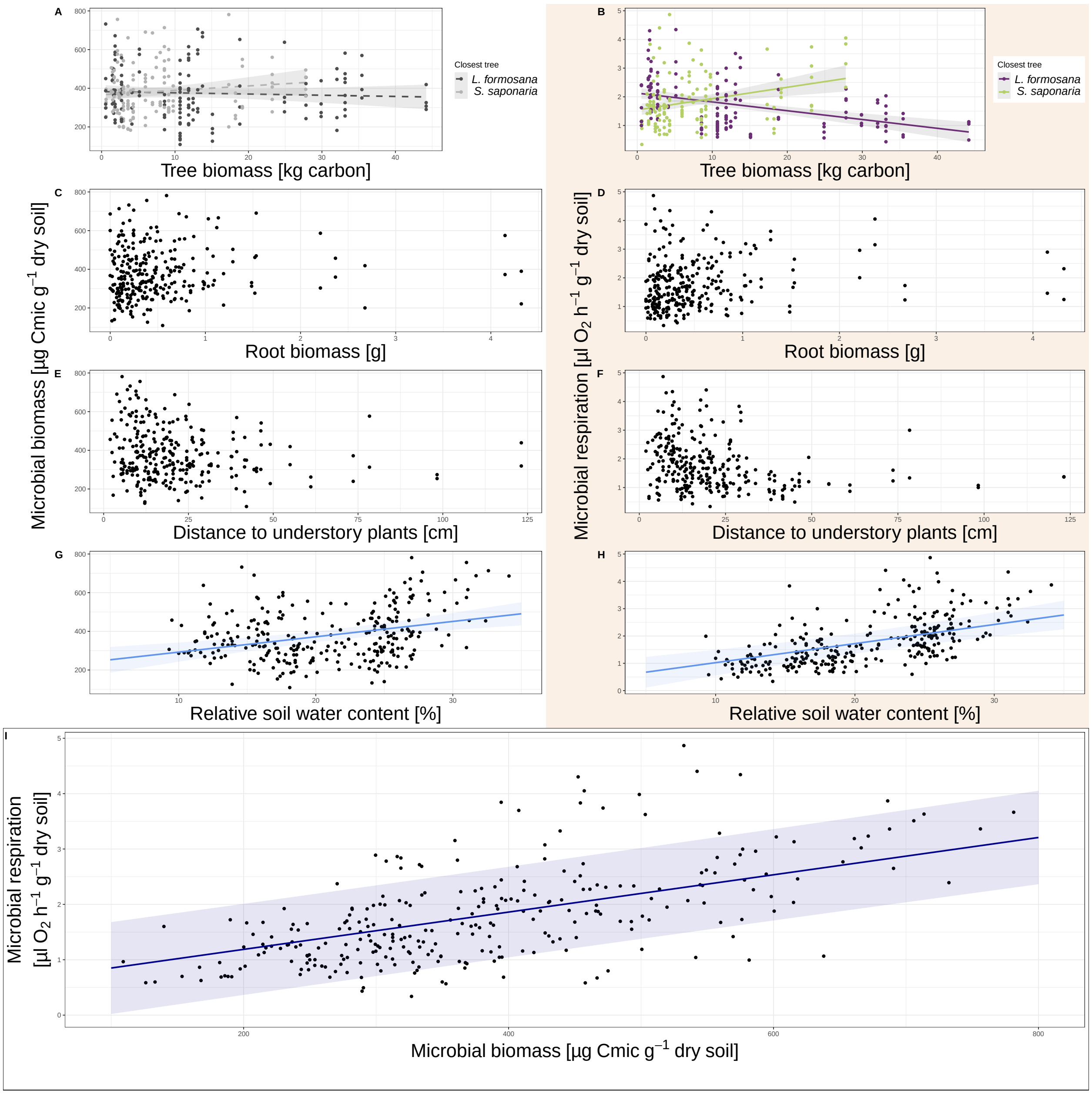

Figure S6: Overview of small scale drivers of microbial biomass and respiration. Effects of tree size and species identity on microbial biomass (A) and respiration (B). Effects of root biomass and distance to understory plants were non-significant on microbial biomass (C, E, respectively) or respiration (D, F, respectively). Positive effect of relative soil water content on microbial biomass (G) and respiration (H). Positive correlation of microbial biomass and respiration (I). Significant effects in colour.
